# Magnetic guided stereotactic radioablation for mid-wall ventricular tachycardia: how to overcome limits of catheter ablation

**DOI:** 10.1093/ehjcr/ytaf158

**Published:** 2025-04-01

**Authors:** Michele Magnocavallo, Pietro Rossi, Domenico Marchesano, Stefano Bianchi

**Affiliations:** Arrhythmology Unit, Isola Tiberina—Gemelli Isola, via di Ponte Quattro Capi 39, 00186 Rome, Italy; Arrhythmology Unit, Isola Tiberina—Gemelli Isola, via di Ponte Quattro Capi 39, 00186 Rome, Italy; Radiation Oncology, San Pietro Fatebenefratelli Hospital, via Cassia 600, 00189 Rome, Italy; Arrhythmology Unit, Isola Tiberina—Gemelli Isola, via di Ponte Quattro Capi 39, 00186 Rome, Italy

## Case description

**Figure 1:**
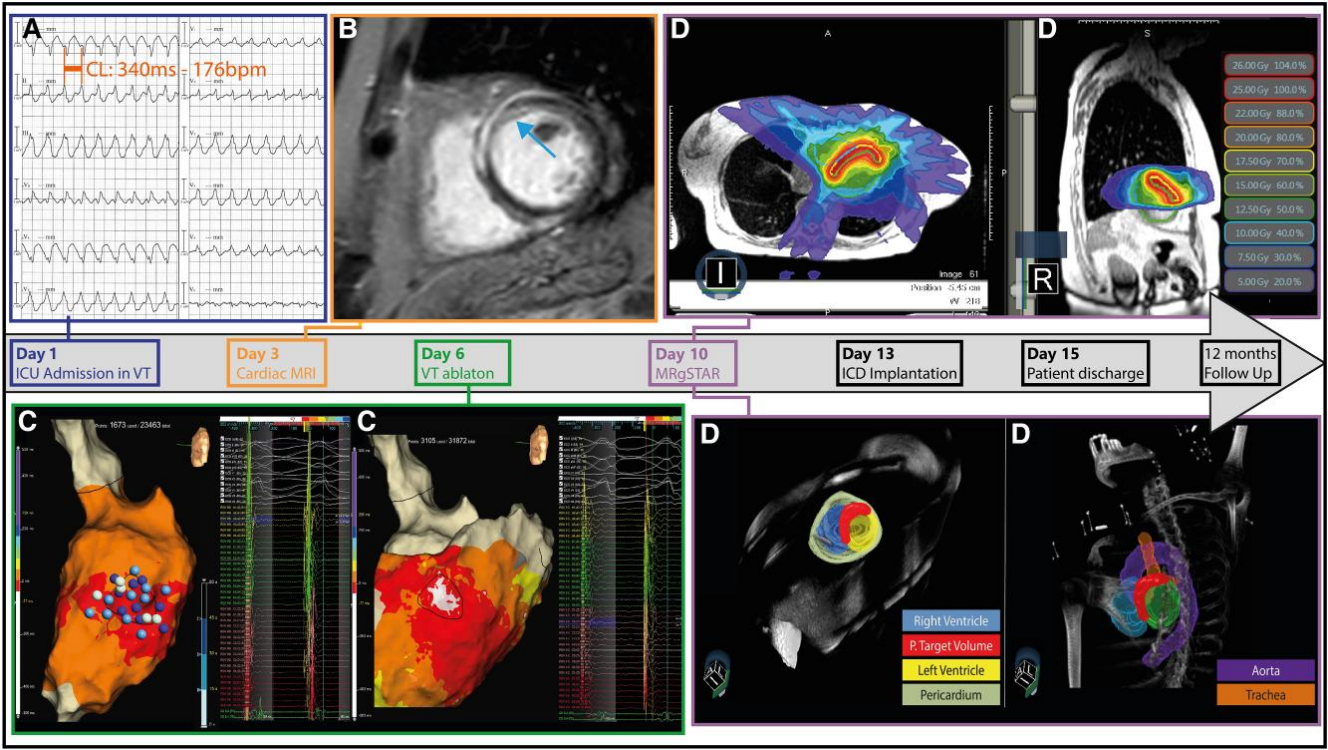
Workflow for magnetic resonance guided sterotactic arrhythmia radioablation (MRgSTAR). Radiotherapy target was achieved by combining electrophysiological [ECG (Panel *A*), electroanatomic maps (Panel *C*—Blue dots represent the ablation lesions)] and anatomical/functional [cardiac MRI—blue arrow showed a basal antero-septal mid-myocardial scar (Panel *B*)] data. A multi-disciplinary team (radiation oncologist, electrophysiologist, and cardiac radiologist) delineated the CTV and planned target volume was obtained, adding a 2–3 mm margin to the target area (Panel *D*). CTV, clinical target volume; MRI, magnetic resonance imaging.

A 64-year-old female was admitted to our intensive care unit for incessant ventricular tachycardia (VT) unresponsive to antiarrhythmic medication (beta-blocker plus amiodarone) (*Figure 1A*). Cardiac magnetic resonance imaging (MRI) revealed a preserved left ventricular function with an ejection fraction of 60% and a basal antero-septal mid-myocardial late gadolinium enhancement (*Figure 1B*). Ultra-high-density left ventricular endo-epicardial maps with the HD grid catheter (Abbott, Minneapolis, USA) performed in sinus rhythm did not show low voltage areas and fragmented/prolonged electrograms; VT activation map identified only the exit site of the tachycardia, and it remained inducible despite multiple radiofrequency applications performed at 50 W using the Tactiflex catheter (*Figure 1C*).

Considering the acute VT recurrence after catheter ablation, the clinical implication of a partial diastolic pathway mapping^1^ and the lack of a well-established strategy for the treatment of arrhythmias originating from mid-wall substrate, we planned a magnetic resonance guided sterotactic arrhythmia radioablation (MRgSTAR). We delineated the clinical target volume (CTV)—antero-septal scar—and the planning target volume was obtained by adding a 2–3 mm margin to the CTV (*Figure 1D*). Radiotherapy energy (25 Gy) was delivered on the CTV, and a sagittal cine MRI was performed during the whole delivery time (see Supplementary material online, *Video S1*). ICD implantation was performed after MRgSTAR and the patient was discharged 2 days later. Genetic test revealed a LMNA gene mutation [c.185G > T;p.(Arg62Leu)] and ICD interrogation at 12-months after MRgSTAR did not show VT recurrence.

STAR was introduced for the treatment of VT refractory to catheter ablation and demonstrated a strong reduction in the arrhythmia burden.^2^ However, no data are available for patients with mid-myocardial scar. In this scenario, radiotherapy energy seems to provide changes in conduction tissue properties and myolisis acutely and after months myocardial fibrosis.^3^ For these reasons, MRgSTAR may represent a safe and effective strategy for the treating of these patients.

## Supplementary Material

ytaf158_Supplementary_Data

## Data Availability

All data are incorporated into the article and its online Supplementary material.

## Lead author biography

Michele Magnocavallo is an EP Fellow from Isola Tiberina Gemelli Isola Hospital. His fields of interests are ventricular tachycardias and prevention of cardiac sudden death. He's involved in the EP Team for the treatment of VT with STAR.
